# Is implant choice associated with fixation strength for displaced radial neck fracture: a network meta-analysis of biomechanical studies

**DOI:** 10.1038/s41598-023-33410-w

**Published:** 2023-04-27

**Authors:** Yu-Cheng Su, Ying-Yu Wang, Ching-Ju Fang, Wei-Ren Su, Fa-Chuan Kuan, Kai-Lan Hsu, Chih-Kai Hong, Min-Long Yeh, Chii-Jeng Lin, Yu-Kang Tu, Chien-An Shih

**Affiliations:** 1 Linkou Chang Gung Memorial Hospital, Taoyuan City, Taiwan; 2 Department of Secretariat, College of Medicine, National Cheng Kung University Hospital, National Cheng Kung University, Tainan, Taiwan; 3 Medical Library, National Cheng Kung University, Tainan, Taiwan; 4 Department of Orthopedics, National Cheng Kung University Hospital, College of Medicine, National Cheng Kung University, Tainan, Taiwan; 5 Medical Device R&D Core Laboratory, National Cheng Kung University Hospital, Tainan, Taiwan; 6 Department of Biomedical Engineering, National Cheng Kung University, Tainan, Taiwan; 7 President Office, Joint Commission of Taiwan, New Taipei City, Taiwan, ROC; 8 Institute of Epidemiology and Preventive Medicine, National Taiwan University College of Public Health, Taipei, Taiwan; 9 Department of Dentistry, National Taiwan University Hospital, Taipei, Taiwan; 10 Department of Orthopedics, College of Medicine, National Cheng Kung University, Tainan, Taiwan

**Keywords:** Biological techniques, Biotechnology, Engineering

## Abstract

The multitude of fixation options for radial neck fractures, such as pins, screws, biodegradable pins and screws, locking plates, and blade plates, has led to a lack of consensus on the optimal implant choice and associated biomechanical properties. This study aims to evaluate the biomechanical strength of various fixation constructs in axial, sagittal, and torsional loading directions. We included biomechanical studies comparing different interventions, such as cross/parallel screws, nonlocking plates with or without augmented screws, fixed angle devices (T or anatomic locking plates or blade plates), and cross pins. A systematic search of MEDLINE (Ovid), Embase, Scopus, and CINAHL EBSCO databases was conducted on September 26th, 2022. Data extraction was carried out by one author and verified by another. A network meta-analysis (NMA) was conducted in accordance with the Preferred Reporting Items for Systematic Reviews and Meta-analyses guidelines. Primary outcomes encompassed axial, bending, and torsional stiffness, while the secondary outcome was bending load to failure. Effect sizes were calculated for continuous outcomes, and relative treatment ranking was measured using the surface under the cumulative ranking curve (SUCRA). Our analysis encompassed eight studies, incorporating 172 specimens. The findings indicated that fixed angle constructs, specifically the anatomic locking plate, demonstrated superior axial stiffness (mean difference [MD]: 23.59 N/mm; 95% CI 8.12–39.06) in comparison to the cross screw. Additionally, the blade plate construct excelled in bending stiffness (MD: 32.37 N/mm; 95% CI − 47.37 to 112.11) relative to the cross screw construct, while the cross-screw construct proved to be the most robust in terms of bending load failure. The parallel screw construct performed optimally in torsional stiffness (MD: 139.39 Nm/degree; 95% CI 0.79–277.98) when compared to the cross screw construct. Lastly, the nonlocking plate, locking T plate, and cross-pin constructs were found to be inferior in most respects to alternative interventions. The NMA indicated that fixed angle devices (blade plate and anatomic locking plate) and screw fixations may exhibit enhanced biomechanical strength in axial and bending directions, whereas cross screws demonstrated reduced torsional stability in comparison to parallel screws. It is imperative for clinicians to consider the application of these findings in constraining forces across various directions during early range of motion exercises, taking into account the distinct biomechanical properties of the respective implants.

## Introduction

Radial neck fractures (RNFs) account for 3–4% of all fractures and one-third of elbow fractures^1,2^. The radial head stabilizes a valgus stress and works as a weight-bearing structure in axial orientation and aids in maintaining elbow stability^3–7^. Stable surgical fixation of displaced RNFs is mandatory to permit early postoperative range of motion exercises, prevent elbow stiffness and restore elbow function^8,9^. Progressive fracture displacement, nonunion, hardware failure and loss of reduction are not uncommon due to inadequate fracture fixation^10–13^.

In contrast to pediatric RNFs, which typically employ pins or elastic nail fixation^14^, adult RNFs present a multitude of fixation options such as metal^13,15–17^ or biodegradable^18,19^ pins/screws, locking or nonlocking plates^13,16^, and blade plates^12,13^. Screw and pin fixation are less invasive approaches that offer low profile fixation and interfragmentary compression, enhancing construct stability and yielding satisfactory outcomes^17^. Plate fixation is also a popular treatment option^16^, but the lack of direction contact between the fracture site may lead to biomechanical inferiority and unfavorable outcomes^20^. Additionally, the biomechanical advantages of fixed-angle devices over nonlocking devices are also unknown for the fixation of RNFs^11,12,21,22^. Currently, there is no consensus on which fixation method provides better fixation strength for displaced radial neck fractures^11–13,16,17,20–23^.

Although there have been several clinical meta-analyses evaluating outcomes of radial head fractures between arthroplasty, resection and interval fixation for adult radial head fractures^24–27^, none have been performed for adult RNF, either biomechanically or clinically. Therefore, the aim of the present study is to perform a systematic review and network meta-analysis (NMA) with an up-to-date search of existing evidence for comparisons of biomechanical properties between different fixation constructs in terms of axial, sagittal and torsional loading.

## Methods

### Search methods for the identification of studies

The NMA was performed according to the preferred reporting items for Systematic Reviews and Meta-analyses (PRISMA) guidelines (Supplementary Table 1)^28^ and was registered at PROSPERO (CRD 42022323386). We searched Embase, Medline and Scopus databases without language restriction until September 26, 2022. The following Medical Subject Heading terms were used: radius, fracture fixation, cadaver, synthetic bone, artificial bone, biomechanic, or mechanic. The complete search strategy and algorithm are shown in Supplementary Table 2. In addition, the reference lists of identified studies were also screened for potentially eligible studies that were not indexed in the databases.

### Inclusion and exclusion criteria

The included studies had a clear description of the specimen type, fracture type, fracture fixation, and mechanical testing protocol and provided extractable biomechanical parameters for comparison. The inclusion criteria were biomechanical studies comparing different RNF fixation methods, such as cross/parallel screws, locking plates (LPs), nonlocking plates (NLPs), NLPs with an augmented screw blade plate and cross pins, using either cadaveric or synthetic radii. Trials were excluded when studies were performed with pathologic, pediatric, and animal models or studies comparing the same techniques (pins or screws) with different designs or implant materials (Supplementary Table 3).

### Study selection

Two authors (YCS and YYW) independently screened all the titles and abstracts according to the selection criteria. Full texts were evaluated after proper screening. If disagreements were noted, a third author (CAS) was involved until the conclusion was made.

### Data extraction and dealing with missing data

One author independently (YCS) extracted the following information: the first author’s name, publication year, design of the study, numbers and type of specimens (cadaveric radii or synthetic radii), fracture model (isolated radial neck or combined radial hand and neck models), implant selection, mechanical testing protocol and biomechanical outcomes (stiffness and failure strength). In studies reporting only medians, we used the median as the means and interquartile ranges/1.35 as the standard deviations^29^. Data extraction was confirmed by a second author (CAS).

### Parameter selection

When a study used different plate thicknesses for biomechanical comparison, the 2.7 mm-thick plate construct was extracted, which is the most common plate type among other studies^13,21^. When a study measured stiffness in cyclic loading or failure loading, we extracted the stiffness value measured during cyclic loading since cyclic stiffness is used in most of the biomechanical studies or as the only measured stiffness value.

### Quality

Methodologic quality was independently assessed by two reviewers (YCS and YYW) using the Cochrane risk of bias tool, including randomization, allocation concealment, blinding, incomplete outcomes, selective reporting, and other sources of bias^30^. A third author (CAS) was consulted for any disagreement.

### Outcome measure

Stiffness measured in the axial, bending, and torsional directions was the primary outcome. Load to failure and torque to failure in different directions were the secondary outcomes.

### Data synthesis

We used spreadsheet software (Excel version 2019, Microsoft, Redmond, WA) for data extraction, and the statistical software STATA was later used (StataCorp. 2017. Stata Statistical Software: Release 15; StataCorp LP College Station, TX) for statistical analysis. For direct comparisons and network meta-analysis, we conducted traditional pairwise meta-analysis to combine direct and indirect evidence. Fixed-effects models were used because of limited study numbers for random-effect model estimation. The *I*^2^ and the Cochrane Q test were calculated in the pairwise meta-analysis for evaluation of heterogeneity. NMA was performed combining both direct and indirect evidence for multiple intervention comparisons. For the assumption of transitivity, we considered that any of the interventions in the network could have been given to any specimen in the network. Potential inconsistency was evaluated by a design-by-treatment model for assessing global inconsistency and loop inconsistency models and node-splitting models for local inconsistency^30,31^. Meta-regression analyses were tested for fracture models in terms of fracture pattern and fracture comminution. We calculated the surface under the cumulative ranking curve (SUCRA) to rank the treatment outcomes for different interventions. The publication bias was evaluated by funnel plots and Egger’s regression plots.

## Result

### Study selection and description

**Figure 1:**
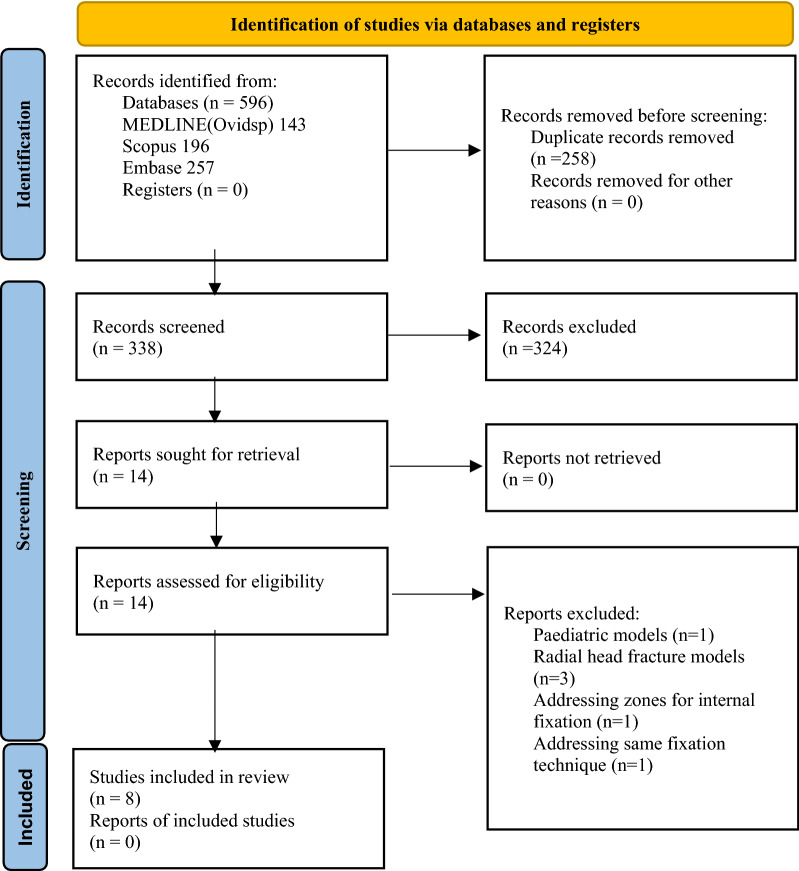
Flowchart of network meta-analysis for biomechanical studies in displaced radial neck fractures.

We identified 338 studies during the study selection process (Fig. 1). After title and abstract screening, 14 biomechanical studies were selected. Ultimately, 8 studies meeting our inclusion and exclusion criteria were eligible for analysis (Table 1)^11–13,16,17,20–22^. These included studies comparing different constructs published from 1900 to 2022 with sample sizes in each group that ranged from 2 to 12 specimens. Three studies involved radial head and neck fractures^12,17,22^, and 5 studies involved radial neck fractures^11,13,16,20,21^. Five studies conducted their experiment with cadaveric radii^11–13,16,21^, while 3 used synthetic radii^17,20,22^. Of the included studies, there were 6 trials using cross-screw fixation^11–13,17,20,22^, 3 trials using LP (T plate) fixation^12,16,20^, 3 trials using LP (anatomic plate) fixation^11,12,22^, 5 trials using non-LP fixation^12,13,16,17,21^, 1 trial using non-LP with augmented screw fixation^16^, 3 trials using blade plate fixation^12,13,21^, 1 trial using parallel screw fixation^20^, and 2 trials using cross-pin fixation^16,17^.

**Table 1:** Characteristics of the included biomechanical studies.

Author	Fracture type	Specimen number	Intervention	Testing protocol	Axial stiffness (N/mm)	Bending stiffness (N/mm)	Torsional stiffness (Nm/degree)	Bending failure load (N)
Burkhart (2007)	RHNF	8/8/8/8/8 Cadaveric radii	NLP BP CS LP(T) LP(A)	A: 5N T: 300 Nmm	NLP: 11.15 (4.23) BP: 19.29 (7.5) CS: 16.07 (10.39) LP(T): 2.41(1.21) LP(A): 39.93 (20.42)	N/A	NLP: 38.76 (14.8) BP: 65.48 (24.21) CS: 13.87 (2.3) LP(T): 15.06 (2.54) LP(A): 106.63 (38.37)	N/A
Capo (2008)	RNF	7/7/7/7 Cadaveric radii	CP LP(T) NLP NLP(AS)	B: 0.04 mm/s (max:50 N) T: 0.5degree/s (max:2000 N)	N/A	CP: 13.58 (3.98) LP(T): 12.27 (2.5) NLP: 12.26 (5.32) NLP(AS): 14.13 (2.87)	CP: 31.09 (13.83) LP(T): 47.81 (22.33) NLP: 53.36 (19.44) NLP(AS): 57.09 (25.79)	N/A
Chen (2017)	RNF	8/8/8 Synthetic radii	LP(T) CS PS	B: 10 N T: 5 degrees/min	N/A	LP(T): 48.73 (6.8) CS: 71.25 (10.88) PS: 67.05 (8.54)	LP(T): 690 (120) CS: 1220 (220) PS: 950 (170)	LP(T): 279.22 (75.36) CS: 418.51 (70.68) PS: 399.73 (81.6)
Giffin (2004)	RNF	2/2/2 Cadaveric radii	BP NLP CS	B: 2 mm/min	N/A	BP: 111.48 (55.23) NLP: 47.12 (15.87) CS: 136.82 (42.9)	N/A	BP: 195.5 (131.5) NLP: 114.5 (9.2) CS: 266.5 (224.2)
Gutowski (2015)	RNF	5/5 Cadaveric radii	LP(A) CS	B: load to failure only	N/A	N/A	N/A	LP(A): 206 (36) CS: 230 (105)
Koslowsky (2007)	RHNF	12/12/12 Synthetic radii	CS NLP CP	B: 50 N	N/A	N/A	N/A	CS: 208.0 (65.9) NLP: 122.7 (40.7) CP: 165.2 (37.9)
Patterson (2001)	RNF	7/7 Cadaveric radii	NLP BP	A: 0.1 mm/sec	NLP: 20.9 (8.5) BP: 36.8 (26)	N/A	N/A	N/A
Rebgetz (2019)	RHNF	10/12 synthetic radii	CS LP (A)	A: 1 mm/min	CS: 659.8 (29.4) LP(A): 678.4 (117)	N/A	N/A	N/A

RHNF, Radial head and neck fracture; RNF, Radial neck fracture; CS, Cross screw; LP(T), Locking plate (T plate); LP (A), Locking plate (anatomic plate); NLP, Nonlocking plate; NLP(AS), Nonlocking plate with augmented screw; BP, Blade plate; PS, Parallel screw; CS, Cross pin; A: Axial load; B: Bending load; T: Torsional load.

*Data are presented as the mean (standard deviation).

### Quality

The main domains for potential bias were the randomization process, allocation concealment, and blinding (Supplementary Table 4). For selection bias, randomization methods were unclear in 5 studies and low in others, and allocation concealment was unclear in all the studies. In all studies selected, performance bias (blinding of participants and personnel) and detection bias (blinding of outcome assessment) were unclear, and the attrition bias (incomplete outcome data) and reporting bias (selective reporting) were low. Other bias was unclear in 3 studies because no cyclic loading was performed and was low in the others.

### Network meta-analysis (combination of direct and indirect comparisons)

**Figure 2:**
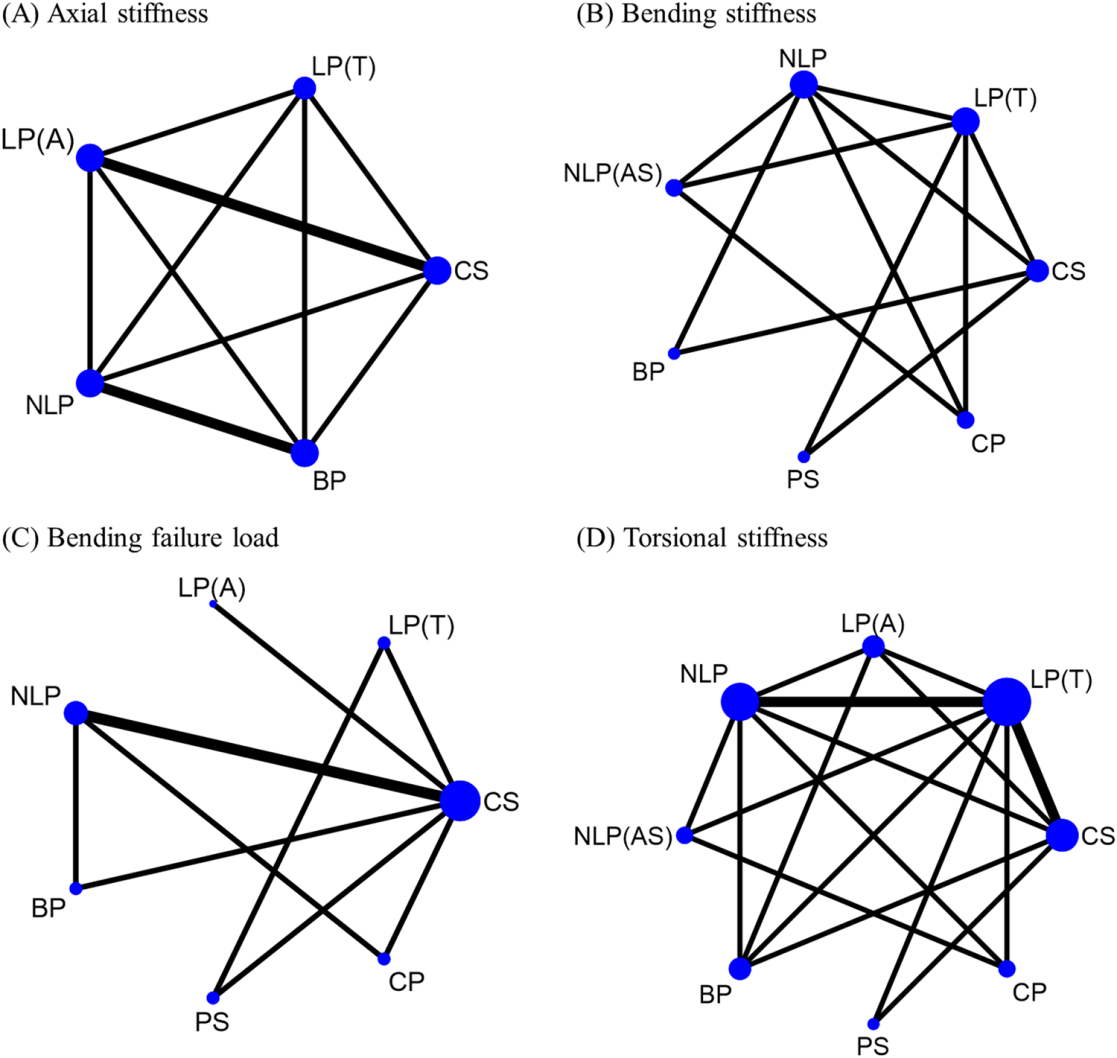
Network of the fixation method for radial neck fractures for (**A**) axial stiffness, (**B**) bending stiffness, (**C**) bending failure load, and (**D**) torsional stiffness. CS, Cross screw; LP(T), Locking plate (T plate); LP(A), Locking plate (anatomic plate); NLP, Nonlocking plate; NLP(AS), Nonlocking plate with augmented screw; BP, Blade plate; PS, Parallel screw; CP, Cross pin.

**Figure 3:**
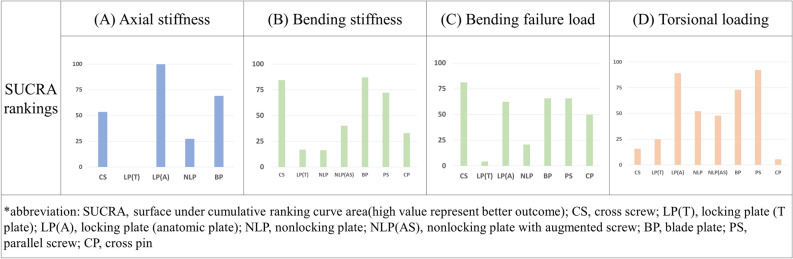
Relative ranking probability of different radial neck fracture fixation methods for (**A**) axial stiffness, (**B**) bending stiffness, (**C**) bending failure load, and (**D**) torsional stiffness. CS, Cross screw; LP(T), Locking plate (T plate); LP(A), Locking plate (anatomic plate); NLP, Nonlocking plate; NLP(AS), Nonlocking plate with augmented screw; BP, Blade plate; PS, Parallel screw; CP, Cross pin.

The network plots for the outcomes of axial stiffness, bending stiffness, torsional stiffness and bending failure load are presented in Fig. 2. The results of the pairwise and network meta-analyses were summarized (Supplementary Table 5) using the summary mean differences (MDs) with 95% CIs**.** The rank probabilities and cumulative probabilities are summarized in Supplementary Fig. 2. The SUCRA-based relative rankings are listed in Fig. 3A–D.

### Axial stiffness

Four studies measured axial stiffness^12,13,21,22^. Compared with the control group (cross screw), the summary MD of axial stiffness was − 13.72 N/mm for the LP-T plate (95% CI − 20.93 to − 6.51), 23.59 N/mm for the LP-anatomic plate (95% CI 8.12 to 39.06), − 5.13 N/mm for the nonlocking plate (95% CI − 12.86 to 2.60), and 3.63 N/mm for the blade plate (95% − 5.13 to 12.40). The LP-anatomic plate (SUCRA = 99.9%) was most likely to be ranked the best in terms of axial stiffness.

### Bending stiffness

Two studies measured bending stiffness^16,20^. Compared with the control group (cross screw), the summary MD of bending stiffness was − 23.78 N/mm for LP-T plate (− 32.57 to − 14.98), − 24.09 N/mm for non-LP (95% CI − 33.87 to − 14.32), − 21.97 N/mm for non-LP with augmented screw (95% CI − 31.20 to − 12.74), 32.37 N/mm for blade plate (95% CI − 47.37 to 112.11), − 5.10 N/mm for parallel screw (95% CI − 14.64 to 4.44) and − 22.52 N/mm for cross pin (95% CI − 31.97 to − 13.07). The blade plate (SUCRA = 87.1%) or cross screw (SUCRA = 84.5%) was most likely to be ranked the best in terms of bending stiffness.

### Bending failure load

Four studies measured the bending failure load^11,13,17,20^. Compared with the control group (cross screw), the summary MD of bending failure load was − 139.29 N for LP-T plate (95% CI − 210.89 to − 67.69), − 24.00 N for LP-Anatomic plate screw (95% CI − 121.29 to 73.29), − 86.60 N for non-LP (95% CI − 129.99 to − 43.20), − 5.71 N for blade plate (95% CI − 193.47 to 182.05), − 18.78 N for parallel screw (95% CI − 93.59 to 56.03), and − 43.74 N for cross pin (95% CI − 86.52 to − 0.96). The cross screw (SUCRA = 81.4%) was most likely to be ranked the best in terms of bending failure load.

### Torsional stiffness

Three studies had biomechanical measurements of torsional stiffness^12,16,20^. Compared with the control group (cross screw), the summary MD of torsional stiffness was 1.19 Nm/degree for LP-T plate (95% CI − 1.18 to 3.56), 92.72 Nm/degree for LP-anatomic plate screw (95% CI 66.08 to 119.35), 21.60 for non-LP (95% CI 12.16 to 31.03), 18.92 Nm/degree for non-LP with augmented screw (95% CI − 3.76 to 41.60), 51.57 Nm/degree for blade plate (95% CI 34.71 to 68.42), 139.39 Nm/degree for parallel screw (95% CI 0.79 to 277.98) and − 7.08 Nm/degree for cross pin (95% CI − 23.03 to 8.86). The parallel screw (SUCRA = 92.3%) or LP-anatomic plate screw (SUCRA = 89.0%) were most likely to be ranked the best in terms of torsional stiffness.

### Reporting bias

Overall, for outcomes regarding axial stiffness, bending stiffness, and bending failure load, the funnel plots showed low publication bias, and Egger’s regression plots did not show any substantial asymmetry. However, there was symmetry, mainly resulting from the comparison between the cross screw and the LP-T plate groups, in the torsional stiffness in the adjusted funnel plots and Egger’s regression plot comparisons (Supplementary Fig. 3).

### Sensitivity analysis

The meta-regression with fracture model and fracture comminution did not moderate axial stiffness and bending failure load outcomes. However, the torsional stiffness would be significantly higher for the LP-T plate group (MD, 521.32 N/mm; 95% CI 349.27–693.37) and significantly higher for the nonlocking plate group (MD, 539.38 N/mm; 95% CI 365.68–713.08) either when using the combined radial head and neck model rather than the isolated radial neck model or when using the comminuted RNF model rather than the noncomminuted model (Supplementary Fig. 4).

### Assessment of inconsistencies

The test for inconsistencies is summarized in Supplementary Table 6. The NMA showed significant global inconsistencies with the design-by-treatment interaction model in bending stiffness, bending strength, torsional stiffness, as well as global inconsistencies with the loop-specific approach in bending stiffness/strength in the loop inconsistency model. NMA on axial stiffness showed no global or local inconsistencies.

The measurement of axial stiffness revealed no significant global inconsistency (*p* = 0.763) or local inconsistency with the loop-specific approach (*p* = 0.4716) or the side-splitting method. For the measurement of bending stiffness, significant global inconsistency (*p* < 0.001) and local inconsistency with the loop-specific approach (*p* = 0.040) and the side-splitting methods (*p* < 0.001) were noted. Regarding the bending failure load measurement, although significant global inconsistency (*p* < 0.001) and local inconsistency using the loop approach (*p* = 0.008) were found, the local inconsistency with the side-splitting method revealed no significant inconsistency (*p* = 0.677). For the measurement of torsional stiffness, NMAs demonstrated significant global inconsistency (*p* < 0.001) and local inconsistency using the side-splitting method; however, local inconsistency using the loop approach revealed no significant inconsistency (*p* = 0.801).

## Discussion

This study represents the first systematic review and network meta-analysis comparing the biomechanical properties of various interventions for displaced radial neck fractures. RNFs, among the most common elbow fractures, typically result from falls on an outstretched arm^32^. Stabilizing the radial head post-fracture is crucial, as it resists valgus stress, serves as a weight-bearing structure in axial orientation, and contributes to elbow stability^3–7^. Excessive force application during the early stages of fracture healing may lead to fixation failure, with axial force generated during daily forearm pronation activities, and bending and torsional forces simulating shear forces applied by the ulna to the radial head^13,20,21^. Therefore, understanding which fixation constructs offer superior biomechanical stability in different directions is essential for informing early post-operative rehabilitation strategies. The NMA revealed that fixed angle constructs, encompassing the anatomic locking plate and blade plate, were most likely to achieve the highest rank in axial stiffness. The blade plate construct demonstrated the greatest performance in bending stiffness, while the cross-screw construct was associated with the optimal outcome in load to failure. In terms of torsional stiffness, the parallel screw construct was found to be the most effective. Conversely, the nonlocking plate, locking T plate, and cross-pin constructs were generally inferior to the majority of other interventions.

Different plate designs may have different biomechanical properties for RNF fixation. In terms of plate fixation for RNFs, our results and those of others^11,22,23^ showed that the anatomic locking plate ranked the best in axial and torsional stiffness. The main advantage of anatomic locking plates for RNF fixation was the anatomical design that fits the proximal radius for better stability by producing higher friction to sustain more axial or torsional loading^12^, resulting from the axial loading of the radial head against the capitellum^21,33^ and the translational forces acting upon the radial head^12^, respectively. Our NMA results also showed that the blade plate outperformed all the other plating constructs in bending stiffness, which was consistent with previous studies^12,13,21^. Since the bending force originated from the shear forces applied to the head by the ulna in the sigmoid notch^12^, it is suggested that the blade plate, as a fixed angle system, could provide higher resistance in sagittal bending than a simple screw plate (nonlocking T plate) construct^13^, as shown in our study and other biomechanical studies^13,21^. In addition, the superiority of the plate may be related to the higher stability of rigid fixation and compression, reducing the risk of osteonecrosis and nonunion^13^. Although several advantages were noted, the main concerns of plate fixation were postoperative forearm rotation loss and a higher potential for hardware removal due to implant irritations^34^.

Screw fixation has been a promising alternative technique for RNF fixation because screws can not only have rigid connections through the internal ends of the fracture but also decrease scarring and hardware irritation due to plate placement beneath the annular ligament^12,13,20^. Although oblique/cross screw fixation was described in the fixation for comminuted RNFs before, it was more easily applicable in axially stable RNFs^10^. The NMA showed that crossed-screw fixation had the best ranking in the posterior bending direction and a lower ranking in the torsional directions, which is consistent with prior biomechanical studies showing that the cross-screw construct had better biomechanical performance in sagittal and axial loading than plate constructs^13,23^. However, in terms of torsional loading, care should be taken when small cross screws are applied (≤ 2 mm in diameter) or in the comminuted RNFs, as the fixation strength may be weaker and pose the risks for loss of fixation during torsional movement^12^. In contrast, Chen et al. suggested that the cross-screw fixation may resist higher torsional stress because two ends of the fracture were stressed in the cross-screw fixation^20^. Gutowski et al. suggested that crossed screws are more suitable for simple transverse RNF fixation^11^.

Our NMA results also showed that parallel fixation had better torsional stiffness than the others. However, parallel-screw fixation was less frequently used clinically and was evaluated and compared in only one biomechanical study^20^. The configuration was suggested to have advantages over the cross-screw fixation by either minimizing the soft tissue exposure from parallel trajectories or avoiding the need for forearm rotation that poses the risks of loss of reduction during the insertion of the screws from opposite entry points^20^. Future studies are needed to compare the clinical outcomes of parallel screw fixation with other fixation techniques.

Clinically, crossed metal^16,17,35^ or biodegradable^18,36,37^ pins are also possible options for RNF fixation. The clinical results of biodegradable pins in the treatment of comminuted radial head and neck fractures are promising^36^. However, our NMA showed that using metal crossed-pin fixation was biomechanically weaker, ranking the worst in torsional stiffness and ranking as inferior in bending loading. Poor performance of pins in torsional stiffness was also found in other biomechanical studies^16,17^. The biodegradable pins were not included in the present NMA. However, several biomechanical studies have shown that the biomechanical strength of biodegradable pins is inferior to that of metal screws^18^ and plates^37^ in RNF fixation. Thus, pinning fixation should be used with caution because the biomechanical strength is lower than that of plate and screw fixations.

The main strength of the current NMA is that it is the first study to perform multiple biomechanical comparisons between different fixation methods for RNFs regarding biomechanical strength. However, the study was subject to several limitations. First, the power of some conclusions regarding outcomes on biomechanical strength would be limited because we had a small number of included trials. Second, heterogeneity and inconsistencies existed in the present study, possibly due to the experimental designs and testing protocols, specimen types (cadaveric/synthetic radii specimens), fracture model type (radial neck/radial head with neck models) and the presence of fracture comminution (comminuted/noncomminuted RNF models). However, we performed meta-regression analysis based on the specimen and fracture type, and the rankings were not changed after adjustment. Finally, we employed a fixed effect model for analysis because the trial numbers in the NMA were inadequate for random effects model estimation.

## Conclusion

The NMA indicated that fixed angle devices (blade plate and anatomic locking plate) and screw fixations may exhibit enhanced biomechanical strength in axial and bending directions, whereas cross screws demonstrated reduced torsional stability in comparison to parallel screws. It is imperative for clinicians to consider the application of these findings in constraining forces across various directions during early range of motion exercises, taking into account the distinct biomechanical properties of the respective implants.

## Supplementary Information

Supplementary Information.

## Data Availability

The datasets used and/or analyzed during the current study available from the corresponding author on reasonable request.
