# Supplementary material for: Is implant choice associated with fixation strength for displaced radial neck fracture: a network meta-analysis of biomechanical studies
Source: Sci Rep. 2023 Apr 27;13:6891. doi: 10.1038/s41598-023-33410-w (PMC10140263; doi:10.1038/s41598-023-33410-w)
Supplement: Supplementary file 1 — Supplementary Information. [file 41598_2023_33410_MOESM1_ESM.docx]

**Appendix**

[Supplementary Table 1: PRISMA 2020 checklist 2](#_Toc114068242)

[Supplementary Table 2: Protocol and Search strategies 6](#_Toc114068243)

[Supplementary Table 3: Excluded studies and reasons 8](#_Toc114068246)

[Supplementary Table 4: Risk of bias assessment 9](#_Toc114068247)

[Supplementary Figure 1: Network forest plots 11](#_Toc114068249)

[Supplementary Figure 2: Relative ranking probability 13](#_Toc114068251)

[Supplementary Table 5: League tables 17](#_Toc114068282)

[Supplementary Figure 3: Publication bias 21](#_Toc114068256)

[Supplementary Figure 4: Meta-regression 25](#_Toc114068260)

[Supplementary Table 6: Inconsistency 28](#_Toc114068269)

[Supplementary Figure 5: Contribution plots 35](#_Toc114068282)

**Supplementary Table 1: PRISMA 2020 checklist**

| **Section and Topic** | **Item #** | **Checklist item** | **Reported on Page #** |
| --- | --- | --- | --- |
| **TITLE** | | |  |
| Title | 1 | Identify the report as a systematic review. | **1**  **4, Introduction (3^rd^ paragraph)** |
| **ABSTRACT** | | |  |
| Abstract | 2 | See the PRISMA 2020 for Abstracts checklist. | **2-3, Abstract section** |
| **INTRODUCTION** | | |  |
| Rationale | 3 | Describe the rationale for the review in the context of existing knowledge. | **4, Introduction(2^nd^ paragraph)** |
| Objectives | 4 | Provide an explicit statement of the objective(s) or question(s) the review addresses. | **4, Introduction(3^rd^ paragraph);**  **Supplementary Table 2** |
| **METHODS** | | |  |
| Eligibility criteria | 5 | Specify the inclusion and exclusion criteria for the review and how studies were grouped for the syntheses. | **5, Method(2^nd^ paragraph)** |
| Information sources | 6 | Specify all databases, registers, websites, organisations, reference lists and other sources searched or consulted to identify studies. Specify the date when each source was last searched or consulted. | **5, Method(1^st^ paragraph)** |
| Search strategy | 7 | Present the full search strategies for all databases, registers and websites, including any filters and limits used. | **5, Method(1^st^ paragraph);**  **Supplementary Table 2.** |
| Selection process | 8 | Specify the methods used to decide whether a study met the inclusion criteria of the review, including how many reviewers screened each record and each report retrieved, whether they worked independently, and if applicable, details of automation tools used in the process. | **5-6, Method(3^rd^paragraph);**  **Table 1;**  **Supplementary Table 3** |
| Data collection process | 9 | Specify the methods used to collect data from reports, including how many reviewers collected data from each report, whether they worked independently, any processes for obtaining or confirming data from study investigators, and if applicable, details of automation tools used in the process. | **6, Method(4^th^ paragraph)** |
| Data items | 10a | List and define all outcomes for which data were sought. Specify whether all results that were compatible with each outcome domain in each study were sought (e.g. for all measures, time points, analyses), and if not, the methods used to decide which results to collect. | **6, Method(5^th^paragraph)** |
|  | 10b | List and define all other variables for which data were sought (e.g. participant and intervention characteristics, funding sources). Describe any assumptions made about any missing or unclear information. | **6, Method(5^th^paragraph)** |
| Study risk of bias assessment | 11 | Specify the methods used to assess risk of bias in the included studies, including details of the tool(s) used, how many reviewers assessed each study and whether they worked independently, and if applicable, details of automation tools used in the process. | **6, Method(6^th^ paragraph)** |
| Effect measures | 12 | Specify for each outcome the effect measure(s) (e.g. risk ratio, mean difference) used in the synthesis or presentation of results. | **6-7, Method(7^th^ paragraph)** |
| Synthesis methods | 13a | Describe the processes used to decide which studies were eligible for each synthesis (e.g. tabulating the study intervention characteristics and comparing against the planned groups for each synthesis (item #5)). | **6, Method(4^th^ paragraph)** |
|  | 13b | Describe any methods required to prepare the data for presentation or synthesis, such as handling of missing summary statistics, or data conversions. | **6, Method(4^th^ paragraph)** |
|  | 13c | Describe any methods used to tabulate or visually display results of individual studies and syntheses. | **7, Method(8^th^ paragraph)** |
|  | 13d | Describe any methods used to synthesize results and provide a rationale for the choice(s). If meta-analysis was performed, describe the model(s), method(s) to identify the presence and extent of statistical heterogeneity, and software package(s) used. | **7, Method(8^th^ paragraph)** |
|  | 13e | Describe any methods used to explore possible causes of heterogeneity among study results (e.g. subgroup analysis, meta-regression). | **7, Method(8^th^ paragraph)** |
|  | 13f | Describe any sensitivity analyses conducted to assess robustness of the synthesized results. | **7, Method(8^th^ paragraph)** |
| Reporting bias assessment | 14 | Describe any methods used to assess risk of bias due to missing results in a synthesis (arising from reporting biases). | **6, Method(6^th^ paragraph)** |
| Certainty assessment | 15 | Describe any methods used to assess certainty (or confidence) in the body of evidence for an outcome. | **N/A** |
| **RESULTS** | | |  |
| Study selection | 16a | Describe the results of the search and selection process, from the number of records identified in the search to the number of studies included in the review, ideally using a flow diagram. | **8, Result(1^st^ paragraph);**  **Figure 1** |
|  | 16b | Cite studies that might appear to meet the inclusion criteria, but which were excluded, and explain why they were excluded. | **8, Result(1^st^ paragraph);**  **Supplementary Table 3** |
| Study characteristics | 17 | Cite each included study and present its characteristics. | **8, Result(1^st^ paragraph);**  **Table 1** |
| Risk of bias in studies | 18 | Present assessments of risk of bias for each included study. | **8, Result(2^nd^ paragraph);**  **Supplementary Table 4** |
| Results of individual studies | 19 | For all outcomes, present, for each study: (a) summary statistics for each group (where appropriate) and (b) an effect estimate and its precision (e.g. confidence/credible interval), ideally using structured tables or plots. | **8, Result(1^st^ paragraph);**  **Table 1** |
| Results of syntheses | 20a | For each synthesis, briefly summarise the characteristics and risk of bias among contributing studies. | **9-11, Result(3^rd^-7^th^ paragraph);**  **Figure 3;**  **Supplementary Figure 2;**  **Supplementary Table 5** |
|  | 20b | Present results of all statistical syntheses conducted. If meta-analysis was done, present for each the summary estimate and its precision (e.g. confidence/credible interval) and measures of statistical heterogeneity. If comparing groups, describe the direction of the effect. | **9, Result(9^th^ paragraph);**  **Supplementary Figure 4-5** |
|  | 20c | Present results of all investigations of possible causes of heterogeneity among study results. | **11, Result(9^th^ paragraph);**  **Supplementary Table 6** |
|  | 20d | Present results of all sensitivity analyses conducted to assess the robustness of the synthesized results. | **11-12, Result(10^nd^ paragraph); Supplementary Figure 4** |
| Reporting biases | 21 | Present assessments of risk of bias due to missing results (arising from reporting biases) for each synthesis assessed. | **10-11, Result(8^th^ paragraph);**  **Supplementary Figure 3** |
| Certainty of evidence | 22 | Present assessments of certainty (or confidence) in the body of evidence for each outcome assessed. | **N/A** |
| **DISCUSSION** | | |  |
| Discussion | 23a | Provide a general interpretation of the results in the context of other evidence. | **13, Discussion(1^st^ paragraph)** |
|  | 23b | Discuss any limitations of the evidence included in the review. | **15-16, Discussion(5^th^ paragraph)** |
|  | 23c | Discuss any limitations of the review processes used. | **15-16, Discussion(5^th^ paragraph)** |
|  | 23d | Discuss implications of the results for practice, policy, and future research. | **16, Discussion, Conclusion section** |
| **OTHER INFORMATION** | | |  |
| Registration and protocol | 24a | Provide registration information for the review, including register name and registration number, or state that the review was not registered. | **5, Method(1^st^ paragraph)** |
|  | 24b | Indicate where the review protocol can be accessed, or state that a protocol was not prepared. | **5, Method(1^st^ paragraph)** |
|  | 24c | Describe and explain any amendments to information provided at registration or in the protocol. | **5, Method(1^st^ paragraph)** |
| Support | 25 | Describe sources of financial or non-financial support for the review, and the role of the funders or sponsors in the review. | **Title page, Declarations section** |
| Competing interests | 26 | Declare any competing interests of review authors. | **Title page, Declarations section** |
| Availability of data, code and other materials | 27 | Report which of the following are publicly available and where they can be found: template data collection forms; data extracted from included studies; data used for all analyses; analytic code; any other materials used in the review. | **Title page, Declarations section** |

*From:*  Page MJ, McKenzie JE, Bossuyt PM, Boutron I, Hoffmann TC, Mulrow CD, et al. The PRISMA 2020 statement: an updated guideline for reporting systematic reviews. BMJ 2021;372:n71. doi: 10.1136/bmj.n71

For more information, visit: <http://www.prisma-statement.org/>

# Supplementary Table 2: Protocol and search strategies (PROSPERO Registration number: CRD42022323386)

**(A) PICOS, inclusion and exclusion criteria**

| Patient | Cadaveric and synthetic radii |
| --- | --- |
| Intervention | Parallel screws, non-locking plates, fixed angle devices (locking T or anatomic plates and blade plate) with or without augmented screw, cross pins. |
| Comparator | The cross-screw technique will be most commonly used as the control group. |
| Outcomes | Construct stiffness and load to failure in different loading directions. |
| Study design | Experimental biomechanical studies |
| Inclusion criteria | 1. Biomechanical comparative studies on radial neck fracture using cadaveric or synthetic radii |
| Exclusion criteria | 1. Pathologic fracture models 2. Pediatric fracture fixation models with pin fixation, isolated lateral or medial epicondylar fracture |

## (B) Search vocabulary

| **Database** | # | Search syntax |
| --- | --- | --- |
| **Embase** | 1 | ((radius* OR elbow*) NEAR/3 (fracture* OR injur*)):ti,ab,kw,de |
|  | 2 | 'radius fracture'/de OR 'distal radius fracture'/exp OR 'elbow fracture'/de OR 'elbow injury'/de |
|  | #1 OR #2 | |
|  | 3 | (((fracture* OR bone*) NEAR/3 fixation*) OR osteosynthes* OR osteo-synthes*):ti,ab,kw,de |
|  | 4 | 'fracture fixation'/exp |
|  | #3 OR #4 | |
|  | 5 | (biomech* OR mechanic*):ti,ab,kw,de |
|  | 6 | 'mechanics'/exp |
|  | #5 OR #6 | |
|  | 7 | (cadaver* OR posthumous OR postmort* OR (post NEXT/1 (humous OR mort*)) OR embalm* OR frozen* OR saw* OR syn* OR fresh-frozen):ti,ab,kw,de |
|  | 8 | cadaver/exp OR 'posthumous care'/de |
|  | 9 | (#1 OR #2) AND (#3 OR #4) AND (#5 OR #6) AND (#7 OR #8) AND [embase]/lim  或  (#1 OR #2) AND (#3 OR #4) AND (#5 OR #6) AND [embase]/lim |
|  |  | (#1 OR #2) AND (#3 OR #4) AND (#5 OR #6) AND [embase]/lim |
|  |  | (#1 OR #2) AND (#3 OR #4) AND (#5 OR #6) AND (#7 OR #8) AND [embase]/lim |
| **MEDLINE (Ovid)** | 1 | ((radius* OR elbow *) ADJ3 (fracture* OR injur*)).mp |
|  | 2 | "Radius Fractures"/ OR "Elbow Fractures"/ OR "elbow Injuries"/ |
|  | (1 OR 2) | |
|  | 3 | (((fracture* OR bone*) ADJ3 fixation*) OR osteosynthes* OR osteo-synthes*).mp |
|  | 4 | exp "Fracture Fixation"/ |
|  | (3 OR 4) | |
|  | 5 | (biomech* OR mechanic*).mp |
|  | 6 | Exp "Mechanics"/ OR exp "Biomechanical Phenomena"/ |
|  | (5 OR 6) | |
|  | 7 | (cadaver* OR posthumous OR postmort* OR (post ADJ1 (humous OR mort*)) OR embalm* OR frozen* OR saw* OR synth*).mp |
|  | 8 | exp cadaver/ |
|  | (7 OR 8) | |
|  | 9 | (1 OR 2) AND (3 OR 4) AND (5 OR 6) AND (7 OR 8)  或  (1 OR 2) AND (3 OR 4) AND (5 OR 6) |
| **Scopus** | 1 | **TITLE-ABS-KEY** ((radius* OR elbow*) W/2 (fracture* OR injur*)) |
|  | 2 | **TITLE-ABS-KEY** (((fracture* OR bone*) W/2 fixation*) OR osteosynthes* OR osteo-synthes*) |
|  | 3 | **TITLE-ABS-KEY** (biomech* OR mechanic*). |
|  | 4 | **TITLE-ABS-KEY** (cadaver* OR posthumous OR postmort* OR (post PRE/0 (humous OR mort*)) OR embalm* OR frozen* OR saw* OR synth*) |
|  | #1 AND #2 AND #3 AND #4 | |

Supplementary Table 3: Excluded studies and reasons

- **Pediatric model**
  - Cao J, Smetana BS, Carry P, et al. A pediatric medial epicondyle fracture study using fresh frozen adult cadavers comparing fracture displacement and loss of terminal elbow extension. *Journal of Pediatric Orthopaedics Part B* 2020; 29(2):149-152.
- **Studies using radial head fracture models**
  - Shi X, Pan T, Wu D, et al. Effect of different orientations of screw fixation for radial head fractures: A biomechanical comparison. *Journal of Orthopaedic Surgery and Research* 2017; 12(1).
  - Burkhart KJ, Nowak TE, Appelmann P, et al. Screw fixation of radial head fractures: Compression screw versus lag screw - A biomechanical comparison. *Injury* 2010; 41(10):1015-1019.
  - Wagner FC, Polossek L, Yilmaz T, et al. Biodegradable magnesium vs. polylactide pins for radial head fracture stabilization: a biomechanical study. *Journal of Shoulder and Elbow Surgery* 2021; 30(2):365-372.
- **Studies regarding the fixation technique**
  - Smith GR, Hotchkiss RN. Radial head and neck fractures: anatomic guidelines for proper placement of internal fixation. *Journal of shoulder and elbow surgery / American Shoulder and Elbow Surgeons ... [et al.]* 1996; 5(2 Pt 1):113-117.
- **Studies comparing the Monteggia-like lesions with same techniques** **(Anatomically shaped locking plates)**
  - Eden L, Frey SP, Gilbert F, et al. Anatomically shaped locking plates for radial head and olecranon fracture fixation in Monteggia-like lesions. *Technology and Health Care* 2020; 28(2):193-201.

| **Study ID** | **Notes for risk of bias assessment** |
| --- | --- |
| Burkhart (2007) | 1. Unclear: allocation concealment, blinding for assessment and outcomes |
| Capo (2008) | 1. Unclear: randomization, allocation concealment, blinding for assessment and outcomes 2. No cyclic loading |
| Chen (2017) | Unclear: randomization, allocation concealment, blinding for assessment and outcomes |
| Giffin (2004) | Unclear: randomization, allocation concealment, blinding for assessment and outcomes |
| Gutowski (2015) | Unclear: randomization, allocation concealment, blinding for assessment and outcomes |
| Koslowsky (2007) | 1. Unclear: randomization, allocation concealment, blinding for assessment and outcomes 2. No cyclic loading |
| Patterson (2001) | 1. Unclear: allocation concealment, blinding for assessment and outcomes 2. Using synthetic specimens |
| Rebgetz (2019) | 1. Unclear: allocation concealment, blinding for assessment and outcomes 2. Using synthetic specimens |

# Supplementary Table 4. Risk of bias assessment

1. **General descriptions**

## (B) Risk of bias assessment for individual domains


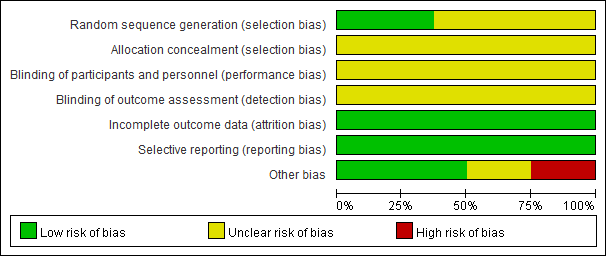


## (C) Risk of bias assessment for individual domains


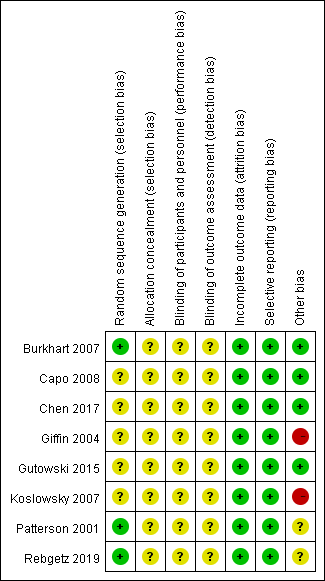


# Supplementary Figure 1: Network forest plots

*Abbreviation: CS, cross screw; LP(T), locking plate (T plate); LP(A), locking plate (anatomic plate); NLP, nonlocking plate; NLP(AS), nonlocking plate with augmented screw; BP, blade plate; PS, parallel screw; CP, cross pin

# (A) Axial stiffness


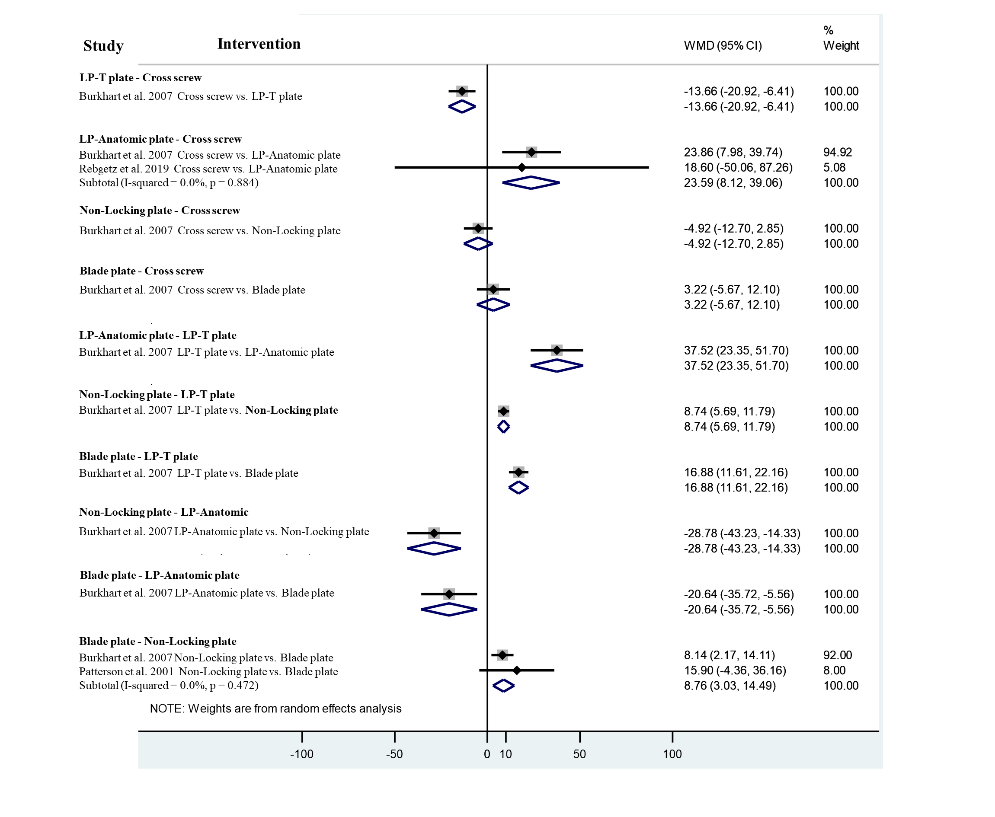


# (B) Bending stiffness


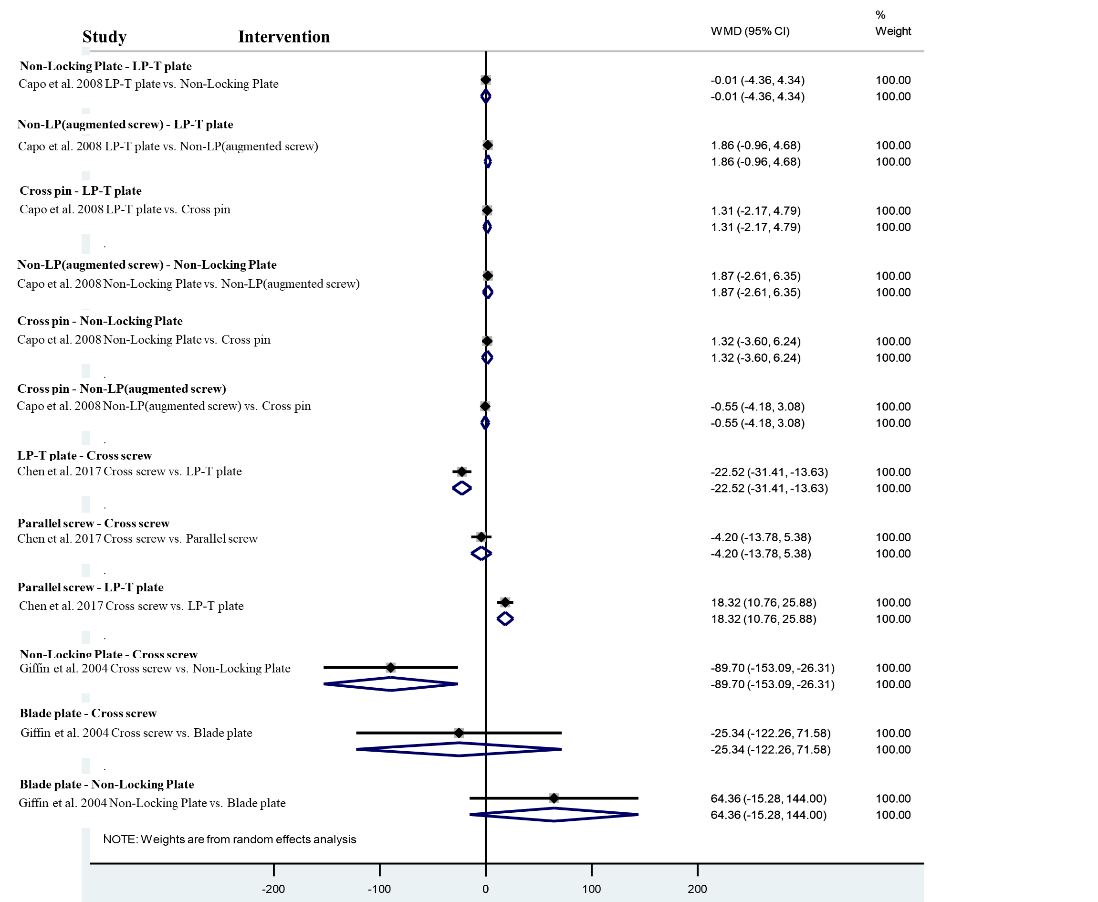


# (C) Bending failure load


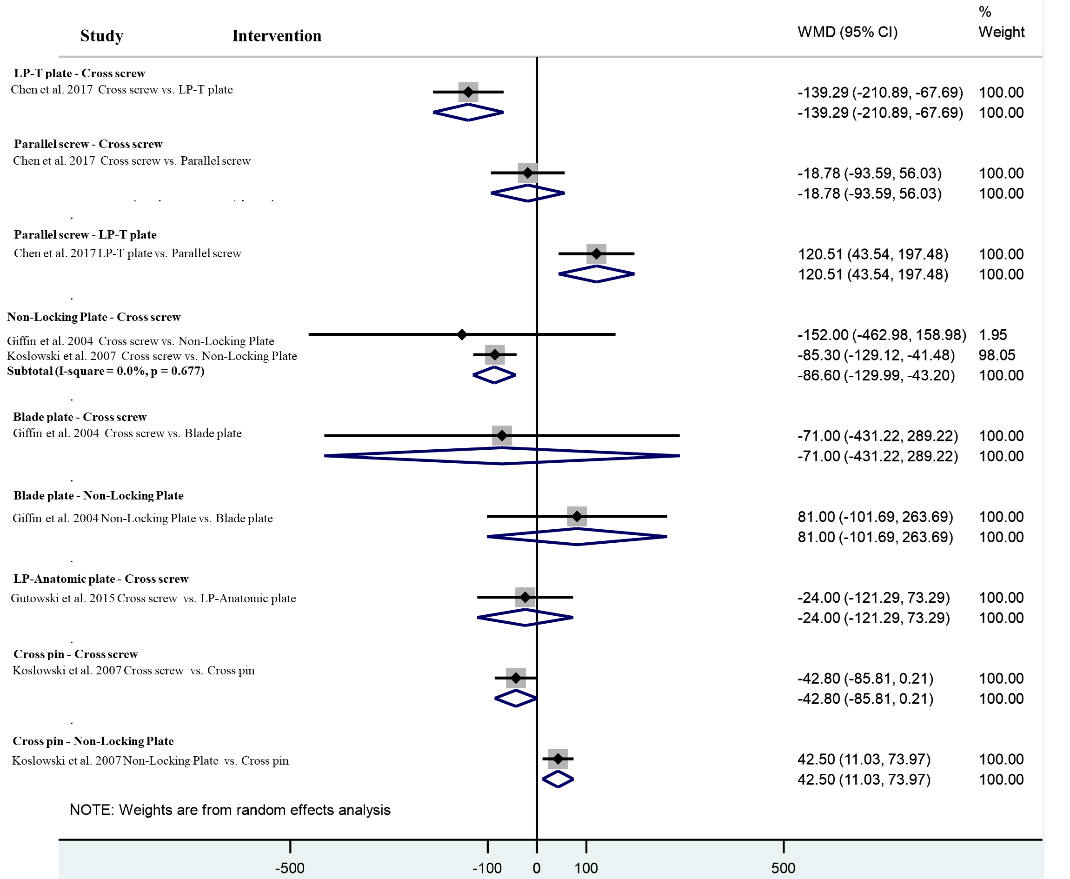


# (D) Torsional stiffness


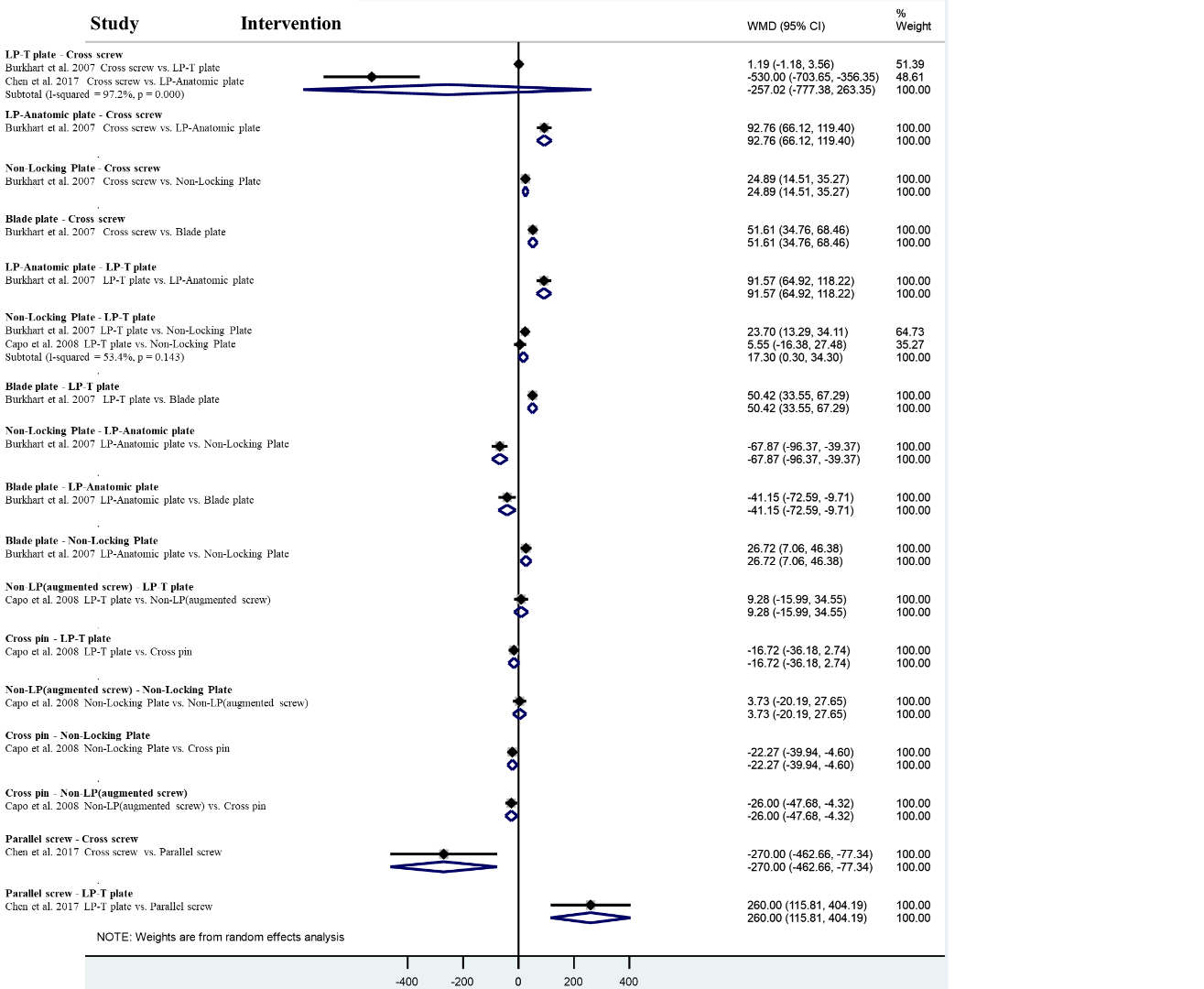


# Supplementary Figure 2. Relative ranking probability

# Axial stiffness

|  | | | | | |
| --- | --- | --- | --- | --- | --- |
| Ranking/constructs | CS | LP(T) | LP(A) | NLP | BP |
| Best | 0.1 | 0.0 | 99.8 | 0.0 | 0.1 |
| 2^nd^ | 23.6 | 0.0 | 0.2 | 0.0 | 76.2 |
| 3^rd^ | 66.8 | 0.0 | 0.0 | 9.5 | 23.7 |
| 4^th^ | 9.5 | 0.0 | 0.0 | 90.5 | 0.0 |
| Worst | 0.0 | 100.0 | 0.0 | 0.0 | 0.0 |
| Mean rank | 2.9 | 5.0 | 1.0 | 3.9 | 2.2 |
| SUCRA | 53.6 | 0.0 | 99.9 | 27.4 | 69.1 |

*Abbreviation: CS, cross screw; LP(T), locking plate (T plate); LP(A), locking plate (anatomic plate); NLP, nonlocking plate; BP, blade plate

## (B) Bending stiffness

|  | | | | | | | |
| --- | --- | --- | --- | --- | --- | --- | --- |
| Ranking/constructs | CS | LP(T) | NLP | NLP(AS) | BP | PS | CP |
| Best | 19.0 | 0.0 | 0.0 | 0.0 | 77.8 | 3.2 | 0.0 |
| 2^nd^ | 68.8 | 0.0 | 0.0 | 0.0 | 4.3 | 26.9 | 0.0 |
| 3^rd^ | 12.2 | 0.6 | 1.0 | 5.1 | 7.6 | 69.9 | 3.6 |
| 4^th^ | 0.0 | 5.2 | 11.8 | 48.3 | 0.9 | 0.0 | 33.8 |
| 5^th^ | 0.0 | 20.2 | 17.1 | 31.3 | 0.3 | 0.0 | 31.1 |
| 6^th^ | 0.0 | 42.5 | 24.2 | 12.5 | 0.8 | 0.0 | 20.0 |
| Worst | 0.0 | 31.5 | 45.9 | 2.8 | 8.3 | 0.0 | 11.5 |
| Mean rank | 1.9 | 6.0 | 6.0 | 4.6 | 1.8 | 2.7 | 5.0 |
| SUCRA | 84.5 | 16.8 | 16.3 | 40.1 | 87.1 | 72.2 | 33.0 |

*Abbreviation: CS, cross screw; LP(T), locking plate (T plate); NLP, nonlocking plate; NLP(AS), nonlocking plate with augmented screw; BP, blade plate; PS, parallel screw; CP, cross pin

## (C) Bending failure load

|  | | | | | | | |
| --- | --- | --- | --- | --- | --- | --- | --- |
| Ranking/constructs | CS | LP(T) | LP(A) | NLP | BP | PS | CP |
| Best | 24.0 | 0.0 | 17.1 | 0.0 | 40.9 | 17.8 | 0.2 |
| 2^nd^ | 45.1 | 0.0 | 18.6 | 0.0 | 10.2 | 21.0 | 5.1 |
| 3^rd^ | 26.3 | 0.3 | 20.6 | 0.1 | 9.1 | 21.5 | 22.1 |
| 4^th^ | 4.6 | 0.6 | 19.9 | 4.0 | 10.1 | 21.8 | 39.0 |
| 5^th^ | 0.0 | 4.0 | 14.3 | 25.6 | 10.6 | 12.5 | 33.0 |
| 6^th^ | 0.0 | 14.8 | 7.8 | 61.5 | 9.9 | 5.4 | 0.6 |
| Worst | 0.0 | 80.3 | 1.7 | 8.8 | 9.2 | 0.0 | 0.0 |
| Mean rank | 2.1 | 6.7 | 3.3 | 5.7 | 3.1 | 3.1 | 4.0 |
| SUCRA | 81.4 | 4.3 | 62.4 | 20.8 | 65.7 | 65.6 | 49.8 |

*Abbreviation: CS, cross screw; LP(T), locking plate (T plate); LP(A), locking plate (anatomic plate); NLP, nonlocking plate; NLP(AS), nonlocking plate with augmented screw; BP, blade plate; PS, parallel screw; CP, cross pin

## (D) Tortional stiffness

|  | | | | | | | | |
| --- | --- | --- | --- | --- | --- | --- | --- | --- |
| Ranking/constructs | CS | LP(T) | LP(A) | NLP | NLP(AS) | BP | PS | CP |
| Best | 0.0 | 0.0 | 23.9 | 0.0 | 0.0 | 0.0 | 76.1 | 0.0 |
| 2^nd^ | 0.0 | 0.0 | 75.4 | 0.0 | 0.1 | 11.7 | 12.8 | 0.0 |
| 3^rd^ | 0.0 | 0.0 | 0.7 | 3.1 | 3.8 | 87.0 | 5.4 | 0.0 |
| 4^th^ | 0.1 | 0.1 | 0.0 | 58.2 | 39.3 | 1.3 | 1.0 | 0.0 |
| 5^th^ | 1.8 | 6.8 | 0.0 | 38.7 | 50.3 | 0.0 | 1.7 | 0.7 |
| 6^th^ | 21.0 | 54.0 | 0.0 | 0.0 | 1.4 | 0.0 | 0.1 | 13.5 |
| 7^th^ | 61.6 | 25.5 | 0.0 | 0.0 | 4.6 | 0.0 | 0.5 | 7.8 |
| Worst | 15.5 | 3.6 | 0.0 | 0.0 | 0.5 | 0.0 | 2.4 | 78.0 |
| Mean rank | 6.9 | 6.3 | 1.8 | 4.4 | 4.6 | 2.9 | 1.5 | 7.6 |
| SUCRA | 15.6 | 24.9 | 89.0 | 52.1 | 47.9 | 72.9 | 92.3 | 5.3 |

*Abbreviation: CS, cross screw; LP(T), locking plate (T plate); LP(A), locking plate (anatomic plate); NLP, nonlocking plate; NLP(AS), nonlocking plate with augmented screw; BP, blade plate; PS, parallel screw; CP, cross pin

# Supplementary Table 5. League tables

- 1. **Summary of pairwise and network meta-analyses of treatment effectiveness for axial stiffness**

|  | Pairwise Meta-analysis | | | | |
| --- | --- | --- | --- | --- | --- |
| Network Meta-analysis | Cross screw | -13.665(-20.918, -6.412) | 23.592(8.121, 39.063) | 3.218(-5.668, 12.104) | 37.524(23.347, 51.701) |
|  | 13.72 (6.51,20.93) | Locking plate (T plate) | 37.524(23.347, 51.701) | 8.742(5.690, 11.794) | 16.883(11.611, 22.155) |
|  | -23.59 (-39.06,-8.12) | -37.31 (-51.20,-23.42) | Locking plate (anatomic plate) | -28.782(-43.234, -14.330) | -20.641(-35.719, -5.563) |
|  | 5.13 (-2.60,12.86) | -8.59 (-11.62,-5.57) | 28.72 (14.56,42.88) | Nonlocking plate | 8.761(3.031, 14.491) |
|  | -3.63 (-12.40,5.13) | -17.35 (-22.47,-12.24) | 19.96 (5.21,34.71) | -8.76 (-14.49,-3.03) | Blade plate |

NOTE. Effect expressed as MD with 95% CI for network meta-analysis or pairwise meta-analysis.

1. **Summary of pairwise and network meta-analyses of treatment effectiveness for bending stiffness**

|  | Pairwise Meta-analysis | | | | | | |
| --- | --- | --- | --- | --- | --- | --- | --- |
| Network Meta-analysis | Cross screw | -22.52  (-31.41, -13.63) | -89.70  (-153.09, -26.31) | - | -25.34  (-122.26, 71.58) | -4.20  (-13.78, 5.38) | - |
|  | 23.78  (14.98,32.57) | Locking plate (T plate) | -0.01  (-4.36, 4.34) | 1.86  (-0.96, 4.68) | - | 18.32  (10.76, 25.89) | 1.31  (-2.17, 4.79) |
|  | 24.09  (14.32,33.87) | 0.32  (-4.03,4.66) | Nonlocking plate | 1.87  (-2.61, 6.35) | 64.36  (-15.28, 144.00) | - | 1.32  (-3.60, 6.24) |
|  | 21.97  (12.74,31.20) | -1.81  (-4.62,1.01) | -2.12  (-6.59,2.35) | Nonlocking plate  with augmented screw | - | - | -0.55(-4.16, 3.09) |
|  | -32.37  (-112.11,47.37) | -56.14  (-135.52,23.23) | -56.46  (-135.75,22.82) | -54.34  (-133.73,25.05) | Blade plate | - | - |
|  | 5.10  (-4.44,14.64) | -18.67  (-26.23,-11.12) | -18.99  (-27.69,-10.29) | -16.87  (-24.93,-8.80) | 37.47  (-42.23,117.17) | Parallel screw | - |
|  | 22.52  (13.07,31.97) | -1.26  (-4.74,2.23) | -1.57  (-6.49,3.34) | 0.55  (-3.08,4.18) | 54.89  (-24.52,134.30) | 17.42  (9.10,25.73) | Cross pin |

NOTE. Effect expressed as MD with 95% CI for network meta-analysis or pairwise meta-analysis.

1. **Summary of pairwise and network meta-analyses of treatment effectiveness for bending failure load**

|  | Pairwise Meta-analysis | | | | | | |
| --- | --- | --- | --- | --- | --- | --- | --- |
| Network Meta-analysis | Cross screw | -139.29  (-210.89, -67.70) | -24.00  (-121.29, 73.29) | -86.60  (-129.99, -43.20) | -71.00  (-431.22, 289.22) | -18.78  (-93.59, 56.03) | -42.80  (-85.81, 0.21) |
|  | 139.29  (67.69,210.89) | Locking plate (T plate) | - | - | - | 120.51  (43.54, 19.48) | - |
|  | 24.00  (-73.29,121.29) | -115.29  (-236.09,5.51) | Locking plate (anatomic plate) | - | - | - | - |
|  | 86.60  (43.20,129.99) | -52.69  (-136.41,31.03) | 62.60  (-43.93,169.13) | Nonlocking plate | 81.00  (-101.69, 263.69) | - | 42.50  (11.03, 73.97) |
|  | 5.71  (-182.05,193.47) | -133.58  (-334.53,67.36) | -18.29  (-229.76,193.18) | -80.89  (-263.58,101.80) | Blade plate | - | - |
|  | 18.78  (-56.03,93.59) | -120.51  (-197.48,-43.54) | -5.22  (-127.95,117.51) | -67.82  (-154.30,18.66) | 13.07  (-189.04,215.18) | Parallel screw | - |
|  | 43.74  (0.96,86.52) | -95.55  (-178.95,-12.15) | 19.74  (-86.55,126.03) | -42.86  (-74.28,-11.44) | 38.03  (-147.34,223.40) | 24.96  (-61.22,111.14) | Cross pin |

NOTE. Effect expressed as MD with 95% CI for network meta-analysis or pairwise meta-analysis.

**(D) Summary of pairwise and network meta-analyses of treatment effectiveness for tortional stiffness**

|  | Pairwise Meta-analysis | | | | | | | | |
| --- | --- | --- | --- | --- | --- | --- | --- | --- | --- |
| Network Meta-analysis | Cross screw | -257.02  (-777.38, 263.35) | 92.76  (66.12, 119.40) | 24.89(14.51, 35.27) |  | 51.61  (34.76, 68.46) | -270.00  (-462.66, -77.34) |  |  |
|  | -1.19  (-3.56,1.18) | Locking plate (T plate) | 91.57  (64.92, 118.22) | 17.30  (0.30, 34.30) | 9.28  (-15.99, 34.55) | 50.42  (33.55, 67.29) | 260.00  (115.80, 404.19) | -16.72  (-36.18, 2.74) |  |
|  | -92.72  (-119.35,-66.08) | -91.53  (-118.18,-64.88) | Locking plate (anatomic plate) | -67.87  (-96.37, -39.37) |  | -41.15  (-72.59, -9.71) |  |  |  |
|  | -21.60  (-31.03,-12.16) | -20.41  (-29.81,-11.01) | 71.12 (42.95,99.29) | Nonlocking plate | 3.73  (-20.20, 27.66) | 26.72  (7.06, 46.38) |  | -22.27  (-39.94, -4.60) |  |
|  | -18.92  (-41.60,3.76) | -17.73  (-40.35,4.89) | 73.80 (38.89,108.71) | 2.68  (-19.67,25.02) | Nonlocking plate  with augmented screw |  |  | -26.00  (-47.68, -4.32) |  |
|  | -51.57  (-68.42,-34.71) | -50.38  (-67.25,-33.51) | 41.15 (9.71,72.59) | -29.97  (-49.15,-10.79) | -32.65  (-60.81,-4.48) | Blade plate |  |  |  |
|  | -139.39  (-277.98,-0.79) | -138.20  (-276.79,0.39) | -46.67 (-187.79,94.45) | -117.79  (-256.69,21.11) | -120.47  (-260.89,19.95) | -87.82  (-227.43,51.79) | Parallel screw |  |  |
|  | 7.08  (-8.86,23.03) | 8.27  (-7.59,24.13) | 99.80 (68.83,130.76) | 28.68 (13.21,44.15) | 26.00 (4.32,47.68) | 58.65 (35.56,81.74) | 146.47 (6.98,285.96) | Cross pin |  |

NOTE. Effect expressed as MD with 95% CI for network meta-analysis or pairwise meta-analysis.

# Supplementary Figure 3. publication bias

## Funnel plot and Egger’s regression test for axial stiffness

*Symbols for abbreviation: A for cross screw; B for locking plate (T plate); C for locking plate (anatomic plate); D for nonlocking plate; E for blade plate

| Egger's test for small-study effects: | |
| --- | --- |
| Std_Eff | Coefficient Std. err. t *P*>\|t\| [95% conf. interval] |
| slope | -.2881377 .4102793 -0.70 0.499 -1.202297 .6260215 |
| bias | .0971751 .1137971 0.85 0.413 -.1563808 .3507309 |
| Test of H0: no small-study effects *p*= 0.413 | |

## (B) Funnel plot and Egger’s regression test for bending stiffness

*Symbols for abbreviation: A for cross screw; B for locking plate (T plate); C for locking plate (anatomic plate); D for nonlocking plate; E for blade plate; F for parallel screw; G for cross pin

| Egger's test for small-study effects: | |
| --- | --- |
| Std_Eff | Coefficient Std. err. t *P*>\|t\| [95% conf. interval] |
| slope | -2.02e-08 1.40e-08 -1.44 0.181 -5.15e-08 1.11e-08 |
| bias | 9.69e-09 5.45e-09 1.78 0.106 -2.44e-09 2.18e-08 |
| Test of H0: no small-study effects *p* = 0.106 | |

## (C) Funnel plot and Egger’s regression test for bending load to failure

*Symbols for abbreviation: A for cross screw; B for locking plate (T plate); C for nonlocking plate; D for nonlocking plate with augmented screw; E for blade plate; F for parallel screw; G for cross pin

| Egger's test for small-study effects: | |
| --- | --- |
| Std_Eff | Coefficient Std. err. t *P*>\|t\| [95% conf. interval] |
| slope | 3.12201 2.289365 1.36 0.210 -2.157274 8.401294 |
| bias | -.1210275 .0746453 -1.62 0.144 -.2931598 .0511049 |
| Test of H0: no small-study effects *p* = 0.144 | |

## (D) Funnel plot and Egger’s regression test for torsional stiffness

*Symbols for abbreviation: A for cross screw; B for locking plate (T plate); C for locking plate (anatomic plate); D for nonlocking plate; E for nonlocking plate with augmented screw; F for blade plate; G for parallel screw; H for cross pin

| Egger's test for small-study effects: | |
| --- | --- |
| Std_Eff | Coefficient Std. err. t *P*>\|t\| [95% conf. interval] |
| slope | 268.024 17.92601 14.95 0.000 230.2035 305.8446 |
| bias | -22.77788 3.815178 -5.97 0.000 -30.8272 -14.72856 |
| Test of H0: no small-study effects *p* = 0.000 | |

# Supplementary Figure 4: Meta-regression

**(A) SUCRA and mean ranks changes of axial stiffness before and after model adjust**

| Covariate/ SUCRA | CS | LP(T) | LP(A) | NLP | BP |
| --- | --- | --- | --- | --- | --- |
| Unadjusted model | 53.6 | 0.0 | 99.9 | 27.4 | 69.1 |
| Fracture model | 53.8 | 0.0 | 99.9 | 27.3 | 69.0 |
| Fracture comminution | 53.8 | 0.0 | 99.9 | 27.3 | 69.0 |

| Covariate/ Mean rank | CS | LP(T) | LP(A) | NLP | BP |
| --- | --- | --- | --- | --- | --- |
| Unadjusted model | 2.9 | 5.0 | 1.0 | 3.9 | 2.2 |
| Fracture model | 2.8 | 5.0 | 1.0 | 3.9 | 2.2 |
| Fracture comminution | 2.8 | 5.0 | 1.0 | 3.9 | 2.2 |

*Abbreviation: CS, cross screw; LP(T), locking plate (T plate); LP(A), locking plate (anatomic plate); NLP, nonlocking plate; BP, blade plate

**(B) SUCRA and mean ranks changes of bending failure load before and after model adjust**

| Covariate/ SUCRA | CS | LP(T) | LP(A) | NLP | BP | PS | CP |
| --- | --- | --- | --- | --- | --- | --- | --- |
| Unadjusted model | 81.4 | 4.3 | 62.4 | 20.8 | 65.7 | 65.6 | 49.8 |
| Fracture model | 80.4 | 15.5 | 62.6 | 25.4 | 50.7 | 65.3 | 50.2 |
| Fracture comminution | 80.4 | 15.5 | 62.6 | 25.4 | 50.7 | 65.3 | 50.2 |

| Covariate/ Mean rank | CS | LP(T) | LP(A) | NLP | BP | PS | CP |
| --- | --- | --- | --- | --- | --- | --- | --- |
| Unadjusted model | 2.1 | 6.7 | 3.3 | 5.7 | 3.1 | 3.1 | 4.0 |
| Fracture model | 2.2 | 6.1 | 3.2 | 5.5 | 4.0 | 3.1 | 4.0 |
| Fracture comminution | 2.2 | 6.1 | 3.2 | 5.5 | 4.0 | 3.1 | 4.0 |

*Abbreviation: CS, cross screw; LP(T), locking plate (T plate); LP(A), locking plate (anatomic plate); NLP, nonlocking plate; BP, blade plate; PS, parallel screw; CP, cross pin

**(C) SUCRA and mean ranks changes of torsional stiffness before and after model adjust**

| Covariate/ SUCRA | CS | LP(T) | LP(A) | NLP | NLP(AS) | BP | PS | CP |
| --- | --- | --- | --- | --- | --- | --- | --- | --- |
| Unadjusted model | 15.6 | 24.9 | 89.0 | 52.1 | 47.9 | 72.9 | 92.3 | 5.3 |
| Fracture model | 45.1 | 54.7 | 99.9 | 71.4 | 14.2 | 85.8 | 28.8 | 0.1 |
| Fracture comminution | 45.1 | 54.7 | 99.9 | 71.4 | 14.2 | 85.8 | 28.8 | 0.1 |

| Covariate/ Mean rank | CS | LP(T) | LP(A) | NLP | NLP(AS) | BP | PS | CP |
| --- | --- | --- | --- | --- | --- | --- | --- | --- |
| Unadjusted model | 6.9 | 6.3 | 1.8 | 4.4 | 4.6 | 2.9 | 1.5 | 7.6 |
| Fracture model | 4.8 | 4.2 | 1.0 | 3.0 | 7.0 | 2.0 | 6.0 | 8.0 |
| Fracture comminution | 4.8 | 4.2 | 1.0 | 3.0 | 7.0 | 2.0 | 6.0 | 8.0 |

*Abbreviation: CS, cross screw; LP(T), locking plate (T plate); LP(A), locking plate (anatomic plate); NLP, nonlocking plate; NLP(AS), nonlocking plate with augmented screw; BP, blade plate; PS, parallel screw; CP, cross pin

# Supplementary Table 6. Inconsistency

## (A) Summary of inconsistency

| Outcome | Fit design-by-treatment interaction model | Explore loop inconsistency |
| --- | --- | --- |
| Axial stiffness | *P*=0.7636 | *P*=0.4716 |
| Bending stiffness | *P*=0.0401 | *P*=0.0401 |
| Bending failure load | *P*=0.0000 | *P*=0.0075 |
| Torsional stiffness | *P*=0.0000 | *P*=0.8013 |

## (B) Inconsistency for axial stiffness

## 1.Inconsistency between direct and indirect evidence

| Side | Direct | | Indirect | | Difference | | |
| --- | --- | --- | --- | --- | --- | --- | --- |
|  | Coef. | Std. Err. | Coef. | Std. Err. | Coef. | Std. Err. | *P*>\|z\| |
| A B * | -13.665 | 3.70033 | -24.15671 | 71.47769 | 10.49171 | 71.75929 | 0.884 |
| A C * | 23.59199 | 7.893455 | 19.32562 | 1091.036 | 4.266367 | 1091.031 | 0.997 |
| A D * | -4.902628 | 3.956816 | -19.99208 | 20.70811 | 15.08946 | 20.63662 | 0.465 |
| B C * | 3.158158 | 4.530708 | 16.52186 | 20.40624 | -13.3637 | 20.64525 | 0.517 |
| B D * | 37.524 | 7.233098 | 27.0062 | 70.81728 | 10.5178 | 71.91174 | 0.884 |
| B E * | 8.742 | 1.557314 | -6.772667 | 21.40216 | 15.51467 | 21.55427 | 0.472 |
| C D * | 16.883 | 2.68988 | 32.40075 | 21.0657 | -15.51775 | 21.55756 | 0.472 |
| C E * | -28.32108 | 7.252954 | -41.68971 | 21.29508 | 13.36863 | 20.64669 | 0.517 |
| D E | -20.72099 | 7.598673 | -5.615532 | 21.00187 | -15.10546 | 20.64965 | 0.464 |

*Symbols for abbreviation: A for cross screw; B for locking plate (T plate); C for locking plate (anatomic plate); D for nonlocking plate; E for blade plate

##

## 2.Loop inconsistency

Multivariate meta-analysis

Variance-covariance matrix = (none)

Method = fixed Number of dimensions = 4

Number of observations = 3

|  | Coefficient Std. err. z *P*>\|z\| [95% conf. interval] |
| --- | --- |
| _y_B  _cons | -13.72004 3.681131 -3.73 0.000 -20.93492 -6.505156 |
| _y_C  _cons | 23.5919 7.89342 2.99 0.003 8.12108 39.06272 |
| _y_D  _cons | -5.127634 3.944832 -1.30 0.194 -12.85936 2.604095 |
| _y_E  _cons | 3.633774 4.47073 0.81 0.416 -5.128697 12.39624 |

## (C) Inconsistency for bending stiffness 1. Inconsistency between direct and indirect evidence

| Side | Direct | | Indirect | | Difference | | |
| --- | --- | --- | --- | --- | --- | --- | --- |
|  | Coef. | Std. Err. | Coef. | Std. Err. | Coef. | Std. Err. | *P*>\|z\| |
| A B * | -22.52 | 4.536166 | -83.25166 | 31.2252 | 60.73166 | 31.55297 | 0.054 |
| A C * | -89.7 | 32.34399 | -22.49537 | 5.047637 | -67.20463 | 32.73549 | 0.040 |
| A E * | -25.34 | 49.4508 | 109.0693 | 55.23595 | -134.4093 | 65.47097 | 0.040 |
| A F * | -4.2 | 4.890118 | -125.6633 | 62.82584 | 121.4633 | 63.10595 | 0.054 |
| B C * | -.0163513 | 2.221683 | -65.1124 | 32.36577 | 65.09605 | 32.44012 | 0.045 |
| B D * | 1.86 | 1.438596 | -119.6033 | 63.09404 | 121.4633 | 63.10595 | 0.054 |
| B F * | 18.32 | 3.859592 | 139.7833 | 63.04075 | -121.4633 | 63.10595 | 0.054 |
| B G * | 1.31 | 1.776449 | -120.1533 | 63.10265 | 121.4633 | 63.10595 | 0.054 |
| C D * | 1.87 | 2.28471 | 136.2793 | 65.38727 | -134.4093 | 65.47097 | 0.040 |
| C E * | 64.36 | 40.63379 | -70.04926 | 73.71458 | 134.4093 | 65.47097 | 0.040 |
| C G * | 1.32 | 2.511198 | 135.7293 | 65.39558 | -134.4093 | 65.47097 | 0.040 |
| D G * | .5 | . | -55.5 | 1.41e+08 | 56 | 1.41e+08 | 1.000 |

*Symbols for abbreviation: A for cross screw; B for locking plate (T plate); C for locking plate (anatomic plate); D for nonlocking plate; E for blade plate; F for parallel screw; G for cross pin

## 2. Loop inconsistency

Multivariate meta-analysis

Variance-covariance matrix = (none)

Method = fixed Number of dimensions = 6

Number of observations = 3

|  | Coefficient Std. err. z *P*>\|z\| [95% conf. interval] |
| --- | --- |
| _y_B  _cons | -23.7752 4.489044 -5.30 0.000 -32.57356 -14.97683 |
| _y_C  _cons | -24.09323 4.98727 -4.83 0.000 -33.8681 -14.31836 |
| _y_D  _cons | -21.96966 4.709922 -4.66 0.000 -31.20094 -12.73839 |
| _y_E  _cons | 32.36935 40.68404 0.80 0.426 -47.36991 112.1086 |
| _y_F  _cons | -5.102615 4.86758 -1.05 0.295 -14.6429 4.437667 |
| _y_G  _cons | -22.51966 4.823856 -4.67 0.000 -31.97425 -13.06508 |

## (D Inconsistency for bending failure load

## 1. Inconsistency between direct and indirect evidence

| Side | Direct | | Indirect | | Difference | | |
| --- | --- | --- | --- | --- | --- | --- | --- |
|  | Coef. | Std. Err. | Coef. | Std. Err. | Coef. | Std. Err. | *P*>\|z\| |
| A B * | 0 | .0000298 | 128 | 2.72e+09 | -128 | 2.72e+09 | 1.000 |
| A C | . | . | . | . | . | . | . |
| A D | . | . | . | . | . | . | . |
| A E * | -71 | 183.7905 | 62.4 | 189.5996 | -133.4 | 320.4689 | 0.677 |
| A F * | 0 | 45.25483 | 96 | 2.15e+09 | -96 | 2.15e+09 | 1.000 |
| A G * | -42.8 | 21.94542 | -176.2 | 318.9582 | 133.4 | 320.4689 | 0.677 |
| B F * | 128 | 45.25483 | 128 | 3.04e+09 | 0 | 3.04e+09 | 1.000 |
| D E * | 81 | 93.21183 | -52.4 | 333.4959 | 133.4 | 320.4689 | 0.677 |
| D G * | 42.5 | 16.05433 | 175.9 | 320.0092 | -133.4 | 320.4689 | 0.677 |

*Symbols for abbreviation: A for cross screw; B for locking plate (T plate); C for nonlocking plate; D for nonlocking plate with augmented screw; E for blade plate; F for parallel screw; G for cross pin

## 2. Design inconsistency

Multivariate meta-analysis

Variance-covariance matrix = (none)

Method = fixed Number of dimensions = 6

Number of observations = 4

|  | Coefficient Std. err. z *P*>\|z\| [95% conf. interval] |
| --- | --- |
| _y_B  _cons | -139.29 36.52874 -3.81 0.000 -210.885 -67.69498 |
| _y_C  _cons | -24 49.64071 -0.48 0.629 -121.294 73.294 |
| _y_D  _cons | -86.59877 22.14062 -3.91 0.000 -129.9936 -43.20396 |
| _y_E  _cons | -5.708714 95.79632 -0.06 0.952 -193.466 182.0486 |
| _y_F  _cons | -18.78 38.16776 -0.49 0.623 -93.58743 56.02743 |
| _y_G  _cons | -43.74016 21.82889 -2.00 0.045 -86.52401 -.9563218 |

## (E) Inconsistency for torsional stiffness

## 1. Inconsistency between direct and indirect evidence for torsional stiffness

| Side | Direct | | Indirect | | Difference | | |
| --- | --- | --- | --- | --- | --- | --- | --- |
|  | Coef. | Std. Err. | Coef. | Std. Err. | Coef. | Std. Err. | *P*>\|z\| |
| A B * | 1.090716 | 1.211373 | 37.45788 | 24.72524 | -36.36717 | 24.76075 | 0.142 |
| A C * | 92.76 | 13.59019 | -950.3837 | 176.0785 | 1043.144 | 175.5607 | 0.000 |
| A D * | 26.60008 | 5.286819 | -29.42912 | 22.84335 | 56.0292 | 24.52089 | 0.022 |
| A F * | 51.61 | 8.598067 | -991.5337 | 175.7636 | 1043.144 | 175.5607 | 0.000 |
| A G * | -270 | 98.29802 | 792.5708 | 129.8782 | -1062.571 | 177.217 | 0.000 |
| B C * | 91.57 | 13.59553 | 76.22393 | 27.99178 | 15.34607 | 24.5342 | 0.532 |
| B D * | 20.3649 | 4.796581 | 1082.936 | 177.2744 | -1062.571 | 177.217 | 0.000 |
| B E * | 9.28 | 12.89381 | 45.68172 | 22.24596 | -36.40172 | 24.7702 | 0.142 |
| B F * | 50.42 | 8.606507 | 35.07393 | 25.93787 | 15.34607 | 24.5342 | 0.532 |
| B G * | 260 | 73.56969 | -802.5708 | 172.0998 | 1062.571 | 177.217 | 0.000 |
| B H * | -16.72 | 9.927579 | 19.68172 | 20.66855 | -36.40172 | 24.7702 | 0.142 |
| C D * | -67.87 | 14.54002 | -104.2793 | 26.74825 | 36.40934 | 24.77048 | 0.142 |
| C F * | -128 | 19.59592 | 0 | 1.52e+09 | -128 | 1.52e+09 | 1.000 |
| D E * | 3.73 | 12.20678 | -32.67934 | 23.38015 | 36.40934 | 24.77048 | 0.142 |
| D F * | 26.72 | 10.03222 | 63.12934 | 24.59069 | -36.40934 | 24.77048 | 0.142 |
| D H * | -22.27 | 9.017305 | -58.67934 | 21.88465 | 36.40934 | 24.77048 | 0.142 |
| E H * | -32 | 11.31371 | 0 | 7.59e+08 | -32 | 7.59e+08 | 1.000 |

*Symbols for abbreviation: A for cross screw; B for locking plate (T plate); C for locking plate (anatomic plate); D for nonlocking plate; E for nonlocking plate with augmented screw; F for blade plate; G for parallel screw; H for cross pin

## 2.Loop inconsistency

Multivariate meta-analysis

Variance-covariance matrix = (none)

Method = fixed Number of dimensions = 7

Number of observations = 3

|  | Coefficient Std. err. z *P*>\|z\| [95% conf. interval] |
| --- | --- |
| _y_B  _cons | 1.186361 1.209622 0.98 0.327 -1.184454 3.557176 |
| _y_C  _cons | 92.71524 13.59019 6.82 0.000 66.07895 119.3515 |
| _y_D  _cons | 21.59581 4.811855 4.49 0.000 12.16475 31.02687 |
| _y_E  _cons | 18.91861 11.57087 1.64 0.102 -3.759882 41.5971 |
| _y_F  _cons | 51.56524 8.598064 6.00 0.000 34.71334 68.41714 |
| _y_G  _cons | 139.3857 70.71513 1.97 0.049 .7865502 277.9848 |
| _y_H  _cons | -7.081391 8.135814 -0.87 0.384 -23.02729 8.864511 |

# Supplementary Figure 5. Contribution plots

## (A) Axial stiffness

*Symbols for abbreviation: A for cross screw; B for locking plate (T plate); C for locking plate (anatomic plate); D for nonlocking Plate; E for blade plate

## (B) Bending stiffness

*Symbols for abbreviation: A for cross screw; B for locking plate (T plate); C for locking plate (anatomic plate); D for nonlocking plate; E for blade plate; F for parallel screw; G for cross pin

## (C) Bending failure load

*Symbols for abbreviation: A for cross screw; B for locking plate (T plate); C for nonlocking plate; D for nonlocking plate with augmented screw; E for blade plate; F for parallel screw; G for cross pin

## (D) Torsional stiffness

*Symbols for abbreviation: A for cross screw; B for locking plate (T plate); C for locking plate (anatomic plate); D for nonlocking plate; E for nonlocking plate with augmented screw; F for blade plate; G for parallel screw; H for cross pin
